# Generating Demand for Alternative Protein in Low- and Middle- Income Countries: Opportunities and Experiences from Nutritious and Sustainable Market Solutions

**DOI:** 10.1016/j.cdnut.2023.101996

**Published:** 2023-09-01

**Authors:** Norah Sadowski, Resham Talwar, Edward F. Fischer, Rowena Merritt

**Affiliations:** 1 Department of Neuroscience, Johns Hopkins School of Medicine, Baltimore, MD, United States; 2 Department of Biomedical Engineering, Johns Hopkins University, Baltimore, MD, United States; 3 Department of Anthropology, Vanderbilt University, Nashville, TN, United States; 4 Centre for Health Services Studies, University of Kent, Canterbury, United Kingdom

**Keywords:** demand, protein transition, alternative proteins, plant-based proteins, healthy diets, sustainable food systems

## Abstract

Protein consumption and the demand for high-value nutritional products is growing rapidly in emerging markets. The projected growth of the alternative protein industry may position it well to meet this demand while addressing environmental sustainability and ethical standards. However, adoption of alternative protein products over traditional animal-sourced proteins is not always a clear choice, with factors such as consumer preferences and habitual behaviors influencing consumer decisions. Insights and considerations associated with generating demand for alternative protein products in low- and middle-income countries (LMIC) were identified through 3 case studies: the OBAASIMA Project in Ghana, the Egg Initiative in Ethiopia, and the World Food Programme Farming Coalition project in Armenia. Key findings emphasize the importance of local sourcing, positive messaging, and integration within existing diets and behaviors. Therefore, these factors will be essential for the adoption of novel alternative protein products in LMIC.

## Introduction

The world’s population is projected to reach 9.7 billion in 2050 from 7.9 billion today [1]. Providing people with enough safe, sustainable, and nutrient-rich food will be one of the biggest obstacles caused by this population expansion. To keep up with changing customer wants and behaviors, the food business has continued to develop, and the industry trends have evolved. A greater emphasis on food quality and nutritional content also responds to shifts in dietary patterns and increasing demand from consumers for more ethically and sustainable sourced products [2,3].

The alternative proteins market is projected to grow from the current 13 million metric tons a year to 97 million metric tons by 2035, reaching a market value of at least USD $290 billion by 2035 [4]. Analysis from high-income countries has identified factors contributing to the rise in popularity of alternative proteins. These factors include potential health benefits of increased intake of plant-based, dietary fiber consumption and consumer concerns regarding adverse health effects of consuming diets high in traditional animal-source protein (e.g., from increased intake of saturated fats) along with increased consumer preferences for environmental sustainability and ethical food production [[5], [6], [7], [8]].

From the standpoint of consumer preferences, food is more than a vehicle for supplying nutritional needs. People make food choices based on cultural norms, personal tastes, and structural constraints, such as income, food availability, and affordability. Changes in diets are often provoked by changes in income as well as health concerns and ethical choices [[9], [10], [11], [12]].

As countries experience rising income levels and urbanization, the demand for protein increases, and consumption in emerging markets is increasing rapidly [13]. Stimulating consumer demand for alternative proteins must be based not only in their presumed needs, but in their social and cultural preferences and behaviors. This perspective attempts to bridge the gap between the 2 and provides insights into the risks and opportunities involved in generating demand, specifically in low- and middle- income countries (LMIC).

## Methods

### Cases

To evaluate risks and effective strategies for stimulating demand for alternative protein products in LMIC, 3 case studies were selected. In all 3, organizations promoting nutritional products conducted formative research to understand preferences and attitudes toward alternative protein and nutritionally dense products, worked across the whole food system to ensure supply and demand were simulated simultaneously, and created marketing campaigns to generate demand. The 3 case studies included in this analysis were the OBAASIMA Project in Ghana, the Egg Initiative project in Ethiopia, and the World Food Programme Farming Coalition project in Armenia. Briefly, the OBAASIMA project aims to make safe and nutritious food products by partnering with local food producers to fortify their products and marketing them with a recognizable trademarked seal [14]. The Egg Initiative aims to increase protein and micronutrient nutrition by the incorporation of egg-based products into everyday diets [15]. Finally, the Farming Coalition project in Armenia aims to increase the production of nutritional food by investing and establishing cooperatives for the local smallholder farmer and creating food value chains to increase availability and access to these locally produced foods [16].

### Research questions

Project leaders from the OBAASIMA Project in Ghana, the Egg Initiative project in Ethiopia, and the World Food Programme Farming Coalition project in Armenia were interviewed and asked questions surrounding 3 major topics based on a conceptual framework of acceptance of alternative proteins: *1*) design and implementation of the project, *2*) marketing strategies, and *3*) approaches to simultaneously generate supply and demand [17]. No incentives were offered to participants to take part in the interviews. In-depth, semistructured qualitative interviews were conducted with project leaders to discuss key elements of project creation and design based around communities and community behaviors, social marketing campaigns and strategies, and supply and demand generation. A list of questions to understand project design, marketing strategies, mechanisms to assess supply and demand, and insights gained from completion of various project phases were sent to project leaders before the interview. A 30 to 40 min qualitative interview based on the aforementioned questions was conducted electronically and recorded for subsequent analysis. The question guide was used as an ‘aide-memoire’ and as a general framework for discussion, ensuring that all themes were covered with the necessary prompts but, at the same time, enabling discussions to be spontaneous, flexible, and responsive to the thoughts and opinions of those being interviewed.

### Data analysis

Transcripts from the recordings were transcribed verbatim for analysis purposes, and publications and reports on each project were also reviewed and subjected to thematic analysis [18]. Briefly, interview transcripts were thoroughly read and reread before coding the patterns of data identified within each individual transcript. The subsequent coded patterns were then analyzed to expose common themes among the interviews. The analysis and identified themes were approved by all authors. The following sections will introduce each topic and the insights and considerations identified from each case study.

## Results

Thematic analysis of interviews and published reports revealed 4 primary themes and strategies that were pivotal to each case, including: *1*) taste and consumer behavior; *2*) marketing and messaging; *3*) simultaneous supply and demand; and *4*) sustainability.

### Taste and consumer behavior

Taste is one well-documented factor that influences consumer behavior [[19], [20], [21]]. It emerged as a key factor within each interview as all 3 project leaders stated that taste was one of the biggest factors when it came to the acceptance of alternative protein and nutritional products. Additionally, each project discovered that familiarity with products that are similar in taste and texture to what is already consumed were more likely to succeed in consumer adoption. Therefore, inclusion of alternative protein or nutritional products into already existing consumer behaviors and meals were key drivers to adoption of alternative proteins.

Each organization in the 3 cases conducted consumer research to understand the social, cultural, and behavioral dynamics in relation to eating habits and preferences. This included research into the types of foods commonly consumed by their target demographic of women of reproductive age and children and the purchasing habits and willingness to buy more nutrient-rich products. As a result of this primary consumer research, each organization then conducted taste testing and consumer focus group interviews to gather feedback from their target audiences. Examples of foods tested by the focus groups included foods that incorporated whole grains or egg powder into recipes common to the region or fortifying snack products commonly consumed by the target demographic. This formative research and feedback from participants resulted in formulation changes to make the products or recipes taste better according to current taste preferences.

### Marketing and messaging

Social marketing is the “process that applies marketing principles and techniques to create, communicate and deliver value in order to influence target audience behaviors that benefit society (public health, safety, the environment, and communities) as well as the target audience” [22]. Social marketing has been used successfully in nutrition studies and programs aimed at changing behavior toward consumption of nutritious foods [[23], [24], [25]]. All organizations utilized a social marketing campaign or conducted social marketing research to identify strategies to effectively generate demand for alternative proteins or nutrient-rich foods. Additionally, each organization included consumer research to identify communities’ social and cultural norms and consumer values. By understanding the consumer’s behavior, values, and perceived barriers limiting the behavior change to adopt new products, each case designed products and strategies that would inspire behavior change in the consumer and overcome the hurdles to adoption, with the ultimate goal of improving nutritional status to alleviate malnutrition in the communities.

To change behavior, each organization utilized positive health education and messaging aimed at improving the consumer’s focus on health and wellbeing. For example, the OBAASIMA project used a promotional campaign which included aspirational and emotionally-driven messages such as “Love yourself. Stay strong for your family” and “Stay Health. Stay Strong.” The Egg Initiative and Farming Coalition projects held health educational sessions to reinforce healthy habits for children such as consuming an egg per day or making healthier versions of the child’s favorite food. Additionally, each organization removed barriers to behavior change adoption. One perceived hurdle is lack of familiarity with the product, and to overcome this, the project directed participants to generate healthier versions of their favorite foods by substituting meat for fruits and vegetables or incorporating egg powder into familiar, prominent household recipes. Another hurdle is the lack of awareness of nutritious products. Each project overcame their respective hurdles by making nutritious foods affordable and easy to identify through their promotional campaigns.

### Simultaneous supply and demand

Demand for alternative proteins and nutrient-dense food is rising in LMIC [13,26]. Additionally, given the increased disruptions within the supply chain, more consumers are looking locally to source their goods [27]. Each organization tried to stimulate both supply and demand simultaneously. To coordinate supply and demand generation, each project utilized the information obtained from their social marketing research around the 4 essential factors of marketing: products, price, place, and promotion.

Each organization focused on incorporation of alternative proteins or fortification into products that were already in demand or familiar to consumers. Next, each project utilized nonperishable and sourcing of food from local food manufacturers, shelf-stable egg powder, or local farmers. This greatly increased accessibility and diversity for more nutritious products by significantly reducing food waste during transport, which helps to keep prices lower and mitigates larger supply chain disruptions. Each project focused on ensuring that the products would be easily accessible and available to the consumer, as this would be a key factor to ensure that there would be long-lasting demand. Finally, each organization was able to then generate demand for the products using strategies such as health promotional campaigns, health education, and utilizing aspirational messaging as identified from their social marketing research as previously described.

### Sustainability

Each organization aimed to improve consumer nutrition in the most sustainable ways possible. They each designed these programs with sustainability in mind from the very beginning by utilizing environmentally sustainable and nutrient-dense products, such as eggs, which have a relatively lower carbon emission compared to other animal products, or by working within existing food systems to bring suppliers and nutritional products more directly to the consumer [28]. The OBAASIMA project partnered with local food manufacturers, such as the Yedent Agro Group in Ghana, to fortify products already popular with consumers, for example the Tom Vita hot breakfast cereal blend made from maize, soybean, and millet. The Egg Initiative project supported individual chicken ownership as well as using egg powder, which is a shelf-stable alternative. Finally, to enhance the likelihood of sustainability the Farming Coalition project in Armenia introduced and promoted circular economy models such as renewable energy solutions, sustainable water and soil management practices, regenerative agriculture. In all cases, improvements such as less food waste, more affordable products, and reports of improved nutrition among the consumers were observed.

## Discussion

Humans have a desire to ingest nutrient-poor food that is high in sugars, fats, and salt [29]. Unfortunately, this means that overcoming this desire requires nutrient-rich foods to also taste good as well as contribute to a healthy lifestyle. The growing population and changing human preferences pose a significant challenge of providing sustainable, nutritious, and delicious food as countries acquire greater wealth [[4], [5], [6], [7], [8]]. To address this challenge, it is crucial to develop tastier and healthier food products and develop effective strategies to directly deliver them to consumers.

Companies that are first to market have the advantage of obtaining key assets such as strategic infrastructure, financial investments, and brand recognition [30]. But supply alone is insufficient, as there must also be a corresponding level of demand. The novelty of new alternative protein products can generate intrigue for consumers, which companies can leverage in their marketing strategies to increase sales [31]. Although both supply and novelty are valuable to entice consumers to try new products, they are not sufficient to generate demand. Demand will not be present without familiarity and desirability of the product [[32], [33]].

The demand for protein and high-value nutritional products in LMIC has increased in step with rising income levels and urbanization [34,35]. However, to address environmental sustainability and ethical standards, the production and demand for alternative protein products over traditional animal-sourced proteins needs to increase. Since consumer decisions are influenced by social norms and individual taste preferences as well as availability and affordability, alternative protein companies wishing to enter the market in LMIC should understand the local context, nutritional needs, and consumer behaviors in LMIC to help shift the market. This can be done by *1*) understanding the community and existing diets and behaviors; *2*) directing marketing strategies to the consumer that focus on consumers’ needs and aspirations; *3*) identifying current market gaps or opportunities to simultaneously generate supply and demand; and *4*) leveraging sustainable solutions [13,17].

## Author contributions

The authors’ responsibilities were as follows – NS, RT, EFF, RM: conceptualized the outline for the paper; RT, NS: conducted the literature review and wrote the first draft of the manuscript; RM: led the submission process; NS: conducted interviews and thematic analysis; RM, EFF: provided input into the analysis and editing of subsequent drafts and added references; and all authors: read and approved the final manuscript.

## Funding

The authors reported no funding received for this study.

## Data availability

Questions and interview transcripts will be made available upon a formal request and approval by the project principal investigators.

## Conflicts of interest

RT, NS, RM, and EFF did not receive any funding from the commercial or private-sector entities for research or consulting and have no conflicts of interest related to the content of this manuscript. RT and NS are voluntarily involved with the Good Food Institute through the Alternative Protein Project at Johns Hopkins but have received no financial funding from the Institute. This research did not involve human subjects; therefore, it was exempt from institutional review board requirements.
